# Benefits of applying a proxy eligibility period when using electronic health records for outcomes research: a simulation study

**DOI:** 10.1186/s13104-015-1217-6

**Published:** 2015-06-09

**Authors:** Tzy-Chyi Yu, Huanxue Zhou

**Affiliations:** Outcomes Research Methods & Analytics, US Health Economics & Outcomes Research, Novartis Pharmaceuticals Corporation, One Health Plaza, East Hanover, NJ 07936 USA; KMK Consulting, Inc., 7, North Tower, 23 Headquarters Plaza, Morristown, NJ 07960 USA

**Keywords:** Outcomes research, Electronic health records, Simulation study, Proxy eligibility period, Missing data, Chronic obstructive pulmonary disease

## Abstract

**Background:**

Electronic health records (EHRs) can provide valuable data for outcomes research. However, unlike administrative claims databases, EHRs lack eligibility tables or a standard way to define the benefit coverage period, which could lead to underreporting of healthcare utilization or outcomes, and could result in surveillance bias. We tested the effect of using a proxy eligibility period (eligibility proxy) when estimating a range of health resource utilization and outcomes parameters under varying degrees of missing encounter data.

**Methods:**

We applied an eligibility proxy to create a benchmark cohort of chronic obstructive pulmonary disease (COPD) patients with 12 months of follow-up, with the assumption of no missing encounter data. The benchmark cohort provided parameter estimates for comparison with 9,000 simulated datasets representing 10–90% of COPD patients (by 10th percentiles) with between 1 and 11 months of continuous missing data. Two analyses, one for datasets using an eligibility proxy and one for those without an eligibility proxy, were performed on the 9,000 datasets to assess estimator performance under increasing levels of missing data. Estimates for each study variable were compared with those from the benchmark dataset, and performance was evaluated using bias, percentage change, and root-mean-square error.

**Results:**

The benchmark dataset contained 6,717 COPD patients, whereas the simulated datasets where the eligibility proxy was applied had between 671 and 6,045 patients depending on the percentage of missing data. Parameter estimates had better performance when an eligibility proxy based on the first and last month of observed activity was applied. This finding was consistent across a range of variables representing patient comorbidities, symptoms, outcomes, health resource utilization, and medications, regardless of the measures of performance used. Without the eligibility proxy, all evaluated parameters were consistently underestimated.

**Conclusion:**

In a large COPD patient population, this study demonstrated that applying an eligibility proxy to EHR data based on the earliest and latest months of recorded activity minimized the impact of missing data in outcomes research and improved the accuracy of parameter estimates by reducing surveillance bias. This approach may address the problem of missing data in a wide range of EHR outcomes studies.

**Electronic supplementary material:**

The online version of this article (doi:10.1186/s13104-015-1217-6) contains supplementary material, which is available to authorized users.

## Background

Health outcomes research has evolved as the variety of available electronic data sources has expanded along with growth in health information technology [1]. Electronic health records (EHRs) are databases that healthcare providers use to record patient-related information to track care. The increasing availability of these EHRs has provided a valuable data source for outcomes research [2, 3]. EHR data can be used in burden-of-illness and clinical outcomes studies, comparative effectiveness research, and health technology assessments. Other studies have used EHRs from multiple locations to gather clinical information about a given study population [2], an application analogous to a chart review.

The number of studies using EHRs in health outcomes research has grown, ranging from only a handful of studies published in 2000 [2] to over 50 studies reporting globally in the years 2010–2011 alone [3]. As healthcare providers in multiple countries adopt EHRs to record patient care, EHRs are becoming an increasingly important data source for outcomes research and for improving healthcare delivery. For example, the US Patient-Centered Outcomes Research Institute (PCORI) has demonstrated its commitment to supporting EHRs in comparative effectiveness research by funding 11 clinical data research networks based on electronic records of large integrated care networks, as well as 18 patient-powered research networks, to collect and curate patient-provided data in a standardized format [4].

In the US, administrative claims databases, which contain information on patient care that payers use to reimburse healthcare providers, are frequently used in health outcomes research. A major strength of EHRs in outcomes research is their ability to capture detailed clinical information not available in claims data, such as symptoms, biometrics, laboratory test results, and prescribed drugs [3, 5, 6]. EHRs can therefore provide a richer source of real-world information than claims data regarding disease severity and symptoms, prognosis, treatment patterns, and outcomes.

Although EHRS have many advantages, several limitations exist. First, EHRs are subject to technological issues that may affect their use for tracking patient care and for research. These issues include the need for technology support such as systems protection and a lack of interoperability between EHRs and other data sources, which limits the ability to link data across EHR databases [5]. Second, EHR data may be erroneous or uninterpretable, and are prone to inconsistencies in provider-entered data. Unlike claims data, where diagnoses, health services performed, and medications are coded in a standardized manner for reimbursement purposes, EHR data vary by provider and by software system used. Some EHR information may also be in the form of text notes, which may require natural language processing (NLP) before being usable for research [5]. Finally, EHR data capture patient information only when care is provided within the healthcare system. The absence of records does not necessarily reflect the absence of an episode of care [5], and could represent an absence of a need for healthcare, or a missing healthcare encounter if the patient received care at a facility that does not contribute data to the EHR system.

When conducting research using retrospective databases, researchers define the period of observation (study period) during which the study population contributes healthcare encounter data. In studies utilizing administrative claims databases (claims data), only population eligible to receive benefits during the study period should be included in research [7], and this is typically done using eligibility tables (plan enrollment information) that list start and end dates for healthcare and drug benefits. Using a continuous eligibility period ensures that the study period falls within the time of the patient’s recorded treatment and that all episodes of care and outcomes in the study period are captured via complete claims records, provided that the patient’s activity was limited to the recording claims source [7]. Unlike claims data, EHRs do not provide eligibility tables, and thus outcomes research using EHRs is subject to the problem of missing data due to underreporting of healthcare utilization or outcomes rendered at facilities that do not contribute data to the EHR system, which could result in surveillance bias.

Our objective was to test the hypothesis that by identifying and applying a proxy eligibility period to EHR dataset analysis, we could increase the likelihood of obtaining complete EHR data on patient care and outcomes. It is possible to identify each patient’s earliest (first) and last month of receiving medical services in an EHR and to then assume that healthcare was continuously received and recorded during that ‘active’ period of EHR reporting (hereafter “eligibility proxy”). Consequently, the resulting estimates for parameters of interest based on the data would be expected to have less bias than parameter estimates that did not use an eligibility proxy. Although missing data and surveillance bias are still concerns in any database, we hypothesized that their impact would be reduced when an eligibility proxy was applied in EHR dataset analysis.

## Methods

### Patient population, EHR database and study design

Chronic obstructive pulmonary disease (COPD) is an irreversible, obstructive lung disease with high morbidity and mortality [8–10]. COPD affects nearly 24 million people in the US and is a leading driver of disease-management costs that impact the US healthcare system [11], thus ensuring a sufficiently large EHR sample size and a policy-relevant population for the present study. We conducted a simulation study in a cohort of patients with COPD identified in a large, US-based EHR to test the effect on parameter estimates of applying an eligibility proxy under varying degrees of missing encounter data for a range of health resource utilization and outcomes.

We conducted multiple simulations of observational data using the Humedica EHR database (An Optum Company, Boston, MA, USA). Humedica’s data acquisition model starts with the providers of care, including many prominent Integrated Delivery Networks. Humedica aggregates EHR data directly from providers, integrating data from multiple disparate EHRs from across the continuum of care, both inpatient and ambulatory. These data capture a comprehensive clinical picture that includes medications, laboratory results, vital signs, physician notes, diagnoses, procedures, demographics, hospitalizations, and outpatient visits. Once aggregated, Humedica normalizes, validates, and statistically deidentifies these data for use in clinical research. The data are certified as deidentified by an independent statistical expert following US Health Insurance Portability and Accountability Act (HIPAA) statistical deidentification rules, and managed according to Humedica’s customer data use agreements. Because this study did not involve the collection, use, or transmittal of individually identifiable data, Institutional Review Board (IRB) review or approval was not required.

### Cohort identification and inclusion criteria for the benchmark dataset

The study period was January 1, 2007 through December 31, 2012. The first month ‘active’ was defined as the earliest month with a recorded healthcare encounter in the EHR dataset that occurred during the study period. The last month ‘active’ was the latest month with recorded activity in the EHR, up to the most recent record on or before December 31, 2012. The index event was the date of the first recorded Global Initiative for Chronic Obstructive Lung Disease (GOLD) COPD stage [GOLD I (mild), GOLD II (moderate), GOLD III (severe), GOLD IV (very severe)] [12]. Key inclusion criteria for the benchmark dataset were: (a) ≥1 EHR with diagnosis of COPD [International Classification of Diseases, Ninth Revision, Clinical Modification (ICD-9-CM) codes 490.xx, 491.xx, 492.xx, or 496.xx], occurring between January 1, 2007 and December 31, 2012; (b) ≥1 EHR indicating GOLD stage; (c) age ≥40 to ≤90 years on the date of first GOLD stage record during the study period. Additional inclusion criteria were: (d) the first (earliest) month active (as of receiving medical services) was before or the same as the month of the index date; and (e) the last month active (as of receiving medical services) was at least 12 months following the month in which the index date occurred. These additional inclusion criteria allowed a proxy eligibility period for each patient to be created based on the first and last months active, under the assumption that all healthcare was continuously received in the healthcare system and recorded in the EHR between the first and last months active.

### Evaluation period and study variables

Data were evaluated for a 12-month period, starting from and including the index date. Patient demographics included age, gender, race, and geographic region. Parameter estimates were developed for study variables of interest, which included COPD symptoms, exacerbations, healthcare utilization, medication, and comorbidities. ICD-9-CM codes were used to determine comorbidities [cardiovascular disease (CVD), including heart failure, stroke, acute myocardial infarction, and peripheral vascular disease; chronic kidney disease; asthma; or depression; (Additional file 1: Table S1)]. COPD symptoms (shortness of breath, cough, wheezing, reduced ability to perform activities) were based on those reported in EHR notes, extracted using NLP, and classified according to Systematized Nomenclature of Medicine–Clinical Terms (SNOMED-CT) [13]. Humedica’s NLP system, the Amplified Chart Extractor (ACE), was developed using vocabulary from the Unified Medical Language System (UMLS). This vocabulary includes multiple medical dictionaries such as the SNOMED-CT, Logical Observation Identifiers Names and Codes (LOINC), and RxNorm, a listing of generic and branded drugs (among others). Using an architecture based on pipelines and standoff annotations as described in the Unstructured Information Management Architecture (UIMA) [14], the ACE is similar to other UIMA based systems such as cTAKES, as previously described in Savova et al. [15].

Exacerbations were defined as any of the following: prescription for steroids and antibiotics on the same date, COPD-related emergency room visit, or COPD-related hospitalization based on discharge diagnosis. Healthcare resource utilization was defined as all-cause hospitalization based on inpatient admissions, all-cause emergency room visits with a unique service date, all-cause office visits with a unique service date, and medications for COPD. Medications of interest included inhaled corticosteroids (ICS), long-acting beta agonists (LABAs), ICS/LABAs, and long-acting muscarinic antagonists (LAMA).

Comorbidities were reported as dichotomous variables, coded as yes/no, and reported as a percentage. COPD symptoms or exacerbations were reported as either dichotomous or continuous variables (coded as frequency per patient and reported as mean event rate and standard deviation), or both. Healthcare resource utilization and medication use during the follow-up period were reported as percentages or event rates.

### Data analysis

The benchmark complete dataset of EHRs for the COPD cohort was created after applying all inclusion criteria, including the proxy eligibility period. The benchmark dataset provided parameter estimates for variables of interest, to be compared with the simulated datasets; it was also used to create simulated datasets with different amounts of missing data.

A total of 9,000 simulated datasets were created and analyzed. The percentage of individuals missing some of their EHR data was set at each 10th percentile from 10 to 90% inclusive (nine levels, each with 1,000 datasets). To create cohorts with missing data, the amount of missing data per individual was randomly set to be between 1 and 11 months (each patient had at least 1 month of data). For example, in the first group of 1,000 datasets, 10% of the patients would be missing some data, and the amount of missing data per patient would be assigned to vary randomly between 1 and 11 months. A similar approach was applied for the remaining eight levels of missing data (that is, 20–90%). Patients were randomly selected via SURVEYSELECT procedure in SAS 9.2 with a simple random sampling method without replacement. In total, there were 25 rounds of selections for each 10th percentile. Also for each patient, a random number was generated from the uniform distribution on the interval [1, 11] by using RANUNI function in SAS 9.2. This random number indicates the number of months with missing data. A preset seed was used for each of the random selections.

For each patient, missing data were in continuous periods and were set for the period from [index date + 12 months − X months] to [index date + 12 months], where X = the number of missing months between 1 and 11. The last active month was then defined as: [month corresponding to the index date + 12 months − X months]. For example, if X = 2, a patient’s records for the period from [index date + 10 months] to [index date + 12 months] were dropped, and the last active month was then defined as: [month corresponding to the index date + 10 months]. For each simulated dataset, the same study variables as in the benchmark dataset were defined and evaluated.

To assess estimator performance under increasing levels of missing data, two analyses, one restricted by eligibility proxy and one unrestricted, were performed on the 9,000 datasets. For the restricted analysis, patients met the inclusion criteria of the first month active occurring before the index date, and the last month active occurring at least 12 months after the index date. Sample size in each dataset was then decreased commensurate with the percentage of assigned missing data, equal to percentiles between 10 and 90%. For the unrestricted analysis, the inclusion criteria of first/last active months were not applied. Thus, the unrestricted analysis assumed everyone had 12 months of follow-up, but in reality 10–90% of patients would have been missing various months of follow-up. We hypothesized that the results of the restricted analysis, for which the eligibility proxy would reduce the amount of missing data, would be less biased than those from the unrestricted analysis.

### Estimate evaluation criteria

One thousand sample datasets were developed for each of the nine levels of percentiles. Estimates for each study variable ($\hat{\theta }$) were compared with those from the benchmark dataset (*θ*), which had no missing data and 12 months of follow-up after the index date. The quality of the parameter estimates (that is, estimate performance) for each variable was evaluated using bias, percentage change, and root-mean-square error (RMSE), and was compared with the quality of estimates for the benchmark dataset. Each of these measures provides a different type of information about estimator performance, as shown by their definitions below.

Bias of an estimator $\hat{\theta }$ of a parameter *θ* was defined as

$$B(\hat{\theta }) = E\left[ {\hat{\theta }} \right] - \theta$$

Variance of an estimator $\hat{\theta }$ of a parameter *θ* was defined as

$$Var\left( {\hat{\theta }} \right) = \sigma_{{\hat{\theta }}}^{2} = E\left[ {\left| {\hat{\theta } - E\left( {\hat{\theta }} \right)} \right|^{2} } \right]$$

Root-mean-square error is the square root of mean squared error (MSE), and denotes the average of the squares of the difference between the estimator and the estimated parameter. RMSE of an estimator $\hat{\theta }$ of a parameter *θ* was defined as

$$RMSE\left( {\hat{\theta }} \right) = \sqrt {\left( {\sigma_{{\hat{\theta }}}^{2} + \left| {B\left( {\hat{\theta }} \right)} \right|^{2} } \right)}$$

All data analyses were conducted in SAS version 9.3 (SAS Institute Inc., Cary, NC, USA).

## Results

### Data characteristics

**Figure 1:**
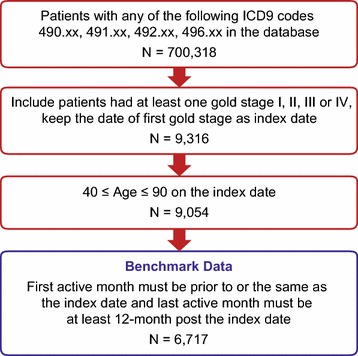
Patient attrition and cohort selection. Inclusion and exclusion criteria show cohort selection for the benchmark data set.

**Figure 2:**
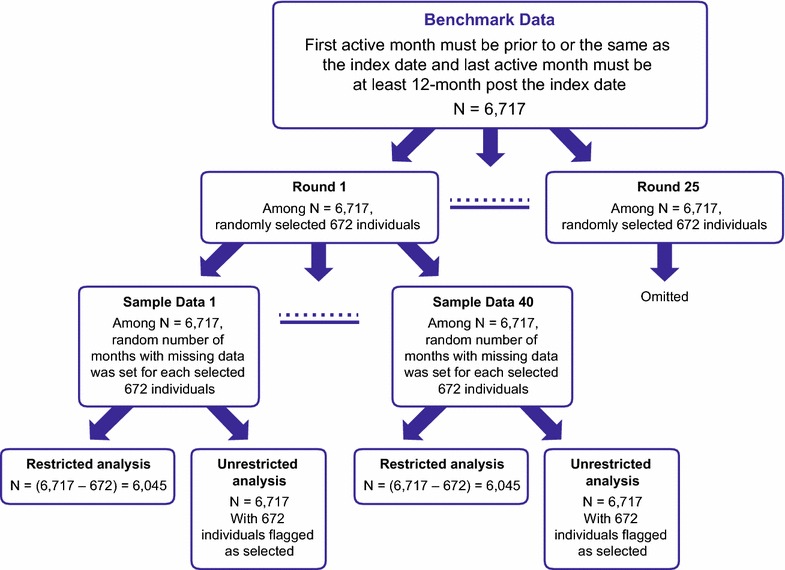
Simulation datasets creating process. An example of how the simulation datasets were derived among random 10% of patients having missing records.

Within the EHR database, 700,318 patients had an ICD-9-CM code for COPD. After applying GOLD stage, age and first/last month active criteria, the final sample size of the benchmark complete dataset was 6,717 (Figure 1). Figure 2 showed the process of creating 1,000 simulated datasets for random 10% of patients having missing records as an example and sample sizes for restricted and unrestricted analysis. The same process was applied to each 10th percentile from 20 to 90%. For the unrestricted analysis, all sample sizes were the same as the benchmark dataset, regardless of the proportion of missing data specified. In the restricted analysis, sample sizes ranged from 6,045 with 10% of patients having missing records to 671 with 90% with missing records (Table 1). In the benchmark dataset, mean age was 69 years (standard deviation (SD) 10.7; Table 2) and median age was 70 years (interquartile range 15 years). Asthma, chronic kidney disease, CVD, and depression were common comorbidities occurring in at least 10% of the sample, and 19.3% of patients had exacerbations within the 12-month follow-up period (Table 2).

**Table 1:** Characteristics of the datasets used in the unrestricted and restricted analyses

Percentage with missing data	Unrestricted analysis		Restricted analysis	
	N	Mean (SD) months of data	N	Mean (SD) months of data
10%	6,717	11.4 (0.01)	6,045	12 (0)
20%	6,717	10.8 (0.02)	5,373	12 (0)
30%	6,717	10.2 (0.02)	4,701	12 (0)
40%	6,717	9.6 (0.02)	4,030	12 (0)
50%	6,717	9.0 (0.03)	3,358	12 (0)
60%	6,717	8.4 (0.03)	2,686	12 (0)
70%	6,717	7.8 (0.03)	2,015	12 (0)
80%	6,717	7.2 (0.04)	1,343	12 (0)
90%	6,717	6.6 (0.04)	671	12 (0)

**Table 2:** Characteristics of benchmark cohort

Variable	N (%)	Mean (SD)
Sample size, final	6,717 (100)	
Age (years)		69 (10.7)
Gender		
Female	3,319 (49.4)	
Male	3,398 (50.6)	
Race		
Caucasian	5,783 (86.1)	
African American	180 (2.7)	
Asian	31 (0.5)	
Other/unknown	723 (10.8)	
US region		
Midwest	3,956 (58.9)	
South	1,529 (22.8)	
West	878 (13.1)	
Northeast	173 (2.6)	
Other/unknown	181 (2.7)	
Outcomes in the 12 months post index		
Comorbidity		
Asthma	1,536 (22.9)	
Chronic kidney disease	840 (12.5)	
Cardiovascular disease	2,186 (32.5)	
Depression	851 (12.7)	
COPD medication of interest		
Inhaled corticosteroid	937 (13.9)	0.21 (0.6)
Inhaled corticosteroid/long-acting beta agonists	2,832 (42.4)	0.72 (1.08)
Long-acting beta agonists	226 (3.4)	0.05 (0.3)
Long-acting muscarinic antagonists	2,219 (33.0)	0.53 (0.93)
COPD-related symptoms		
Cough	3,958 (58.9)	2.93 (5.49)
Reduced ability to perform activities	2,166 (32.2)	1.07 (3.13)
Shortness of breath	4,284 (63.8)	3.48 (5.48)
Wheezing	2,931 (43.6)	1.47 (3.11)
Exacerbations	1,296 (19.3)	0.3 (0.78)
All-cause resource utilization		
Emergency room visit	583 (8.7)	0.17 (0.78)
Hospitalization	596 (8.9)	0.15 (0.56)
Office visit	5,464 (81.3)	9.5 (11.12)

### Bias with missing data

**Figure 3:**
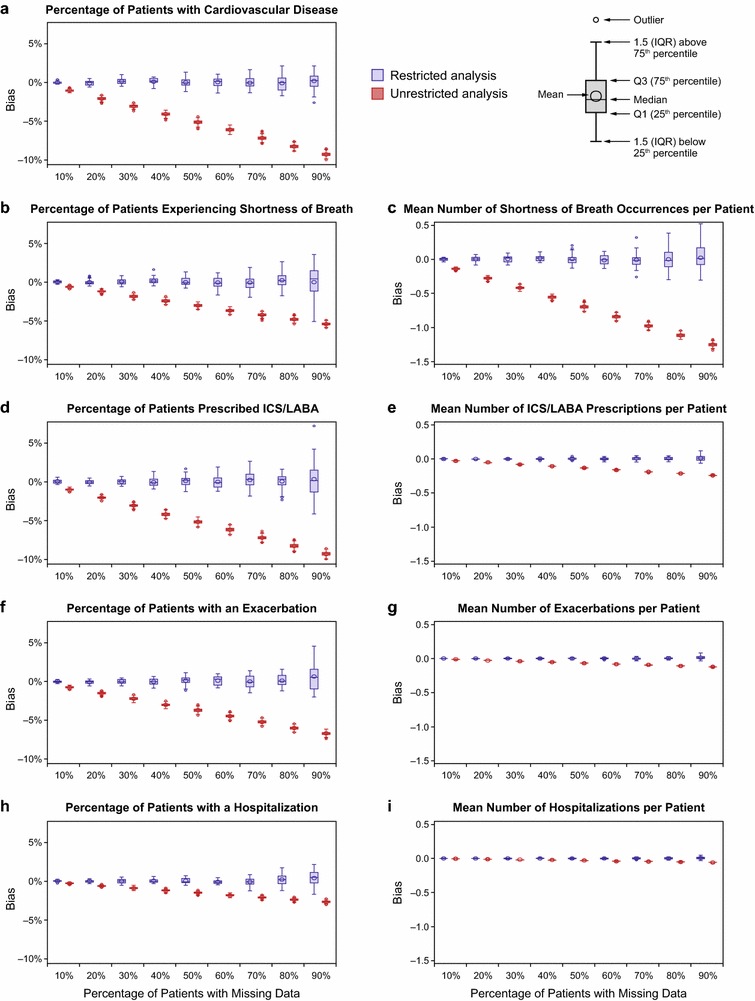
Bias in parameter estimates. Box plots of the bias in selected parameter estimates by percentage of individuals with missing data for the restricted analysis and the unrestricted analysis. **a** Percentage of patients with cardiovascular disease, **b** percentage of patients experiencing shortness of breath, **c** mean number of shortness of breath occurrences per patient, **d** percentage of patients prescribed ICS/LABA, **e** mean number of ICS/LABA prescriptions per patient, **f** percentage of patients with an exacerbation, **g** mean number of exacerbations per patient, **h** percentage of patients with a hospitalization, and **i** mean number of hospitalizations per patient.

In the restricted analysis, in which inclusion criteria for first/last month active were applied, parameter estimates showed very small or almost no bias compared with benchmark data for each variable investigated (Figure 3). However, the variance (spread) in bias increased (y-axis) due to a decrease in the restricted sample size as the percentage of individuals with missing data increased from 10 to 90% (x-axis). In comparison, for the unrestricted analysis, where the first/last active month requirement was not applied, all parameter estimates were systematically underestimated, as shown by an increase in the (negative) magnitude of bias (Figure 3). The differences between estimates from the unrestricted and restricted analyses became more pronounced as the percentage of patients with missing data increased, as shown on the graphs by the increasing separation in bias between the two sets of results.

### Specific examples of the consequences of missing data

The consequences of increased bias also were demonstrated in several examples using different patient descriptors and outcomes. The first was the percentage of patients with CVD, which was 32.5% in the benchmark dataset. Among those with CVD, mean bias was similar in the restricted analysis when evaluated for 10, 50, and 90% of patients missing data, with mean bias of <0.1, 0, and 0.2%, respectively (Figure 3a). In contrast, for the unrestricted analysis, mean bias increased with increasing amounts of missing data (−1, −5.1, and −9.3% corresponding to 10, 50, and 90% of patients missing data, respectively).

The second example pertained to individuals with shortness of breath: 63.8% of the cohort had ≥1 occurrence in the 12-month follow-up period (mean 3.48 occurrences per person; SD 5.48). In the restricted analysis, mean bias in the estimated percentage of individuals with an occurrence was <0.1% in samples with 10% as well as with 90% of individuals having missing data; in the unrestricted analysis, mean bias was −0.6% for the 10% missing data cohorts and increased in magnitude to −5.4% for those with 90% missing data (Figure 3b). For the mean number of occurrences of shortness of breath per patient, mean bias in the restricted analysis was 0.004 and 0.026 in samples with 10 and 90% of individuals missing data (respectively); in the unrestricted analysis, the corresponding values were −0.138 and −1.25 (Figure 3c).

Bias patterns similar to those for CVD and shortness of breath were seen for the percentage of patients with ICS/LABA use (Figure 3d) and with an exacerbation (Figure 3f). Mean bias in the restricted analysis varied little according to the amount of missing data, while mean bias in the unrestricted analysis was larger and increased with the percentage of missing data. With respect to frequency of ICS/LABA use (Figure 3e) or exacerbations (Figure 3g), both of which were relatively rare, differences in bias were smaller between the unrestricted and restricted analyses.

The final example examined bias in an even rarer event, all-cause hospitalization. In the benchmark dataset, 8.9% of the population had ≥1 hospitalization, with a mean of 0.15 (SD 0.56) hospitalizations per patient during the 12-month follow-up period. For the dataset with 10% of patients missing data, the mean bias in the estimated proportion of individuals hospitalized at least once was <0.1% in the restricted analysis and −0.29% in the unrestricted analysis (Figure 3h). For this same dataset, bias in the mean number of hospitalizations per patient was 0 for the restricted analysis and −0.006 for the unrestricted analysis (Figure 3i). In the dataset with 90% of individuals missing records, the bias in the percentage of individuals with a hospitalization was 0.4% for the restricted analysis and −2.7% for the unrestricted analysis. Bias in the estimated mean number of hospitalizations per patient was 0.004 for the restricted analysis and −0.057 for the unrestricted analysis. The magnitude of the bias for rare events, such as the percentage with a hospitalization or the mean number of hospitalizations per patient, was smaller than for more prevalent variables such as the percentage with shortness of breath (Fig. 3b, c) or with ICS/LABA use (Fig. 3d, e).

Bias provides one measure of estimate performance. With respect to the unrestricted analysis, the observed low bias for hospitalization frequency estimates with 90% missing data did not necessarily mean that outcome estimates for rare events (such as hospitalization) were more accurate than those for more frequent events. Therefore, we also assessed estimate performance using other measures, such as percentage change and RMSE.

### Percentage change and RMSE with missing data

**Figure 4:**
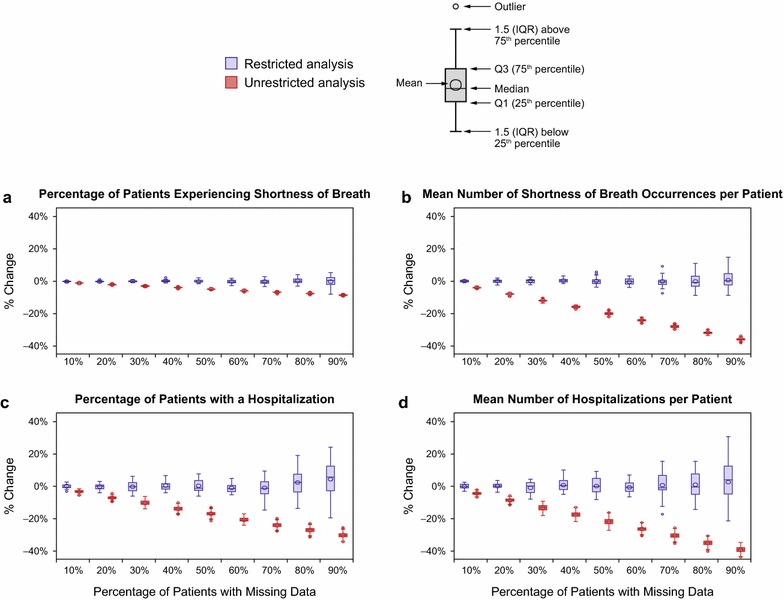
Percentage change in parameter estimates. Box plots of percentage change in selected parameter estimates by percentage of patients with missing data for the restricted analysis and the unrestricted analysis. **a** Percentage of patients experiencing shortness of breath, **b** mean number of shortness of breath occurrences per patient, **c** percentage of patients with a hospitalization, and **d** mean number of hospitalizations per patient.

Additional information about the performance of parameter estimates was provided by using percentage change (Figure 4) and RMSE (Additional file 2: Figure S1). The percentage change between parameter estimates in the benchmark dataset and missing datasets for rare events was larger than for more frequently occurring events. For the frequently occurring event of at least one episode of shortness of breath (63.8% in the benchmark dataset), the percentage change observed in the datasets with 90% of patients missing some data from benchmark estimates was relatively small, at 0.01% in the restricted analysis and −8.47% in the unrestricted analysis (Figure 4a). In contrast, for patients with ≥1 hospitalization, a relatively rare event (8.9% in the benchmark dataset), the percentage change in the estimate for the 90% missing dataset from that for the benchmark dataset was 4.40% in the restricted analysis and −30.14% in the unrestricted analysis (Figure 4c).

For frequency variables, when the magnitude of the estimate was small (as for rare events), such as for the mean number of occurrences of shortness of breath per patient, the percentage change in the parameter estimates in the unrestricted analysis was large. In the dataset with 90% of patients missing data, the percentage change from the benchmark dataset in the mean number of shortness of breath episodes per patient was 0.73% for the restricted analysis and −35.87% for the unrestricted analysis (Figure 4b). The percentage change in mean number of hospitalizations per patient, also a rare event, showed a similar increase from the benchmark dataset in the unrestricted analysis as the percentage of patients missing data increased (Figure 4d). RMSE for parameter estimates demonstrated patterns similar to those for percentage change (Additional file 2: Figure S1).

## Discussion

Using a cohort of patients with COPD identified in a large EHR, we created multiple simulated datasets of COPD patients with differing amounts of missing data to assess the utility of a proxy eligibility period. We found that parameter estimates had better performance when an eligibility proxy based on the first and last month of observed database activity (restricted analysis) was applied. This finding was consistent across a range of variables including patient comorbidities, symptoms, outcomes, health resource utilization, and medications, regardless of the measures of performance used (that is, bias, percentage change, and RMSE). For the unrestricted analysis, in which the eligibility proxy was not applied, all evaluated parameters were consistently underestimated.

In the unrestricted analysis, bias consistently increased as the proportion of patients with missing data increased, yet for many variables evaluated in the unrestricted analysis, the magnitude of bias was relatively low. For dichotomous variables (reported as percentages), bias was often less than 5%, and for continuous variables (reported as frequencies), bias was often less than one unit, even with up to 90% of patients missing some data. Consequently, the minimal observed bias can be a misleading indicator of estimate performance. Estimates using other performance measures, such as percentage change from benchmark estimates, showed that performance could still be poor even if bias was low. These findings illustrate that more than one measure is needed to evaluate estimate performance because different measures reflect different types of performance. For example, bias is particularly sensitive to the number of events and thus can be problematic for describing estimate performance for rare events. This is because a rare event occurring during the missing data period reflects a greater proportion of missing data than a more frequent event, resulting in a greater percentage of underreporting than for a more common event.

This study is unique in that it provides an easily implemented method to minimize the impact of missing data and to improve the accuracy of parameter estimates when using EHR data in outcomes research. EHRs are attractive for outcomes research because, in addition to healthcare and drug utilization and outcomes data, they provide useful information not available in many administrative claims databases, such as biometrics, symptoms, and laboratory results. However, unlike claims databases, EHRs lack eligibility tables (plan enrollment information) and thus are subject to surveillance bias due to missing data. Researchers can address the problem of missing data by applying an eligibility proxy based on the earliest and latest months of recorded activity in the EHR.

This study is limited by assumptions made when applying the eligibility proxy and by features of EHR datasets. It was assumed that records within the eligibility period identified by the eligibility proxy (first/last month active) were complete. That is, all medical services a patient received during this time period identified by the eligibility proxy were recorded in EHR datasets. In addition, the accuracy and completeness of the EHRs used in this study are not known. Events may not have been recorded if individuals were not asked about these specifically, or if providers did not document these when reported. Some diagnoses and treatments may have been coded incorrectly. Although prescriptions for medications may have been documented, it is not known whether the prescriptions were filled or taken. Moreover, the number of refills may not have been noted. A limitation inherent to EHR data is the potential for incomplete information, particularly if healthcare was received outside the EHR system. Missing encounter data (including underreporting by patients or providers) between the first month active and the last month active may have resulted in systematic underestimation of events and healthcare resource utilization in the benchmark dataset. This limitation would apply to any EHR study and is not unique to this US-based EHR dataset used in this study.

## Conclusion

In this simulation study of a COPD cohort identified in a large US-based EHR, we demonstrated the benefits of applying an eligibility proxy using information readily available in the EHR (first and last month of observed activity) when conducting outcomes research with these types of data. The simulation showed that, when an eligibility proxy was applied and the analysis was restricted to patients with sufficient follow-up time based on this proxy, estimates for a range of variables of interest had very low or no discernible bias. The benefit of applying an eligibility proxy became more evident as the percentage of missing data increased. When possible, researchers using EHRs should include a proxy eligibility period to increase the likelihood of having a complete EHR for the study period, in turn reducing surveillance bias.

## Additional files

**Additional file 1: Table S1.** Table of the ICD-9 CM codes for comorbidities of interest.
**Additional file 2: Figure S1.** Root-mean-square error in parameter estimates. Box plots of root-mean-square error in selected parameter estimates by percentage of individuals with missing data for the restricted analysis and the unrestricted analysis. Panel a. Percentage of patients with cardiovascular disease, b. Percentage of patients experiencing shortness of breath, c. Mean number of shortness of breath occurrences per patient, d. Percentage of patients prescribed ICS/LABA, e. Mean number of ICS/LABA prescriptions per patient, f. Percentage of patients with an exacerbation, g. Mean number of exacerbations per patient, h. Percentage of patients with a hospitalization, and i. Mean number of hospitalizations per patient.  

## Abbreviations

ACE: Amplified Chart Extractor
COPD: chronic obstructive pulmonary disease
CVD: cardiovascular disease
EHR: electronic health record
GOLD: Global Initiative for Chronic Obstructive Lung Disease
ICD-9-CM: International Classification of Diseases, Ninth Revision, Clinical Modification
ICS: inhaled corticosteroid
LABA: long-acting beta agonists
LAMA: long-acting muscarinic antagonist
LOINC: Logical Observation Identifiers Names and Codes
MSE: mean squared error
NLP: natural language processing
PCORI: Patient-Centered Outcomes Research Institute
RMSE: root-mean-square error
SNOMED–CT: Systematized Nomenclature of Medicine–Clinical Terms
UIMA: Unstructured Information Management Architecture
UMLS: Unified Medical Language System
US: United States
