# Erratum to: Benefits of applying a proxy eligibility period when using electronic health records for outcomes research: a simulation study

**DOI:** 10.1186/s13104-015-1300-z

**Published:** 2015-08-11

**Authors:** Tzy-Chyi Yu, Huanxue Zhou

**Affiliations:** Outcomes Research Methods & Analytics, US Health Economics & Outcomes Research, Novartis Pharmaceuticals Corporation, One Health Plaza, East Hanover, NJ 07936 USA; KMK Consulting, Inc., 7, North Tower, 23 Headquarters Plaza, Morristown, NJ 07960 USA

## Erratum to: BMC Res Notes (2015) 8:229 DOI 10.1186/s13104-015-1217-6

After publication of the original article [1], Additional file 1 was updated with the following statement, located towards the end of the document:

“Reproduced with permission from: Mannino DM, Higuchi K, Yu T-C, Zhou H, Li Y, Tian H, et al. Economic burden of chronic obstructive pulmonary disease by presence of comorbidities. CHEST Journal. Published online February 12, 2015. doi:10.1378/chest.14-2434.”

This is to credit an earlier publication in which the same additional file was used. The updated additional file (Additional file 1) can be found below.

## Additional file

Additional file 1:
**Table S1.** Table of the ICD-9 CM codes for comorbidities of interest.

## References

[1] Yu TC, Zhou H (2015). Benefits of applying a proxy eligibility period when using electronic health records for outcomes research: a simulation study. BMC Res Notes.

