# Supplementary material for: Benefits of applying a proxy eligibility period when using electronic health records for outcomes research: a simulation study
Source: BMC Res Notes. 2015 Jun 9;8:229. doi: 10.1186/s13104-015-1217-6 (PMC4467672; doi:10.1186/s13104-015-1217-6)
Supplement: Supplementary file 1 — Additional file 1: Table S1. Table of the ICD-9 CM codes for comorbidities of interest. [file 13104_2015_1217_MOESM1_ESM.pdf]

Appendix 1. ICD-9_CM codes for comorbidities of interest

Table. ICD-9-CM Codes for Comorbidities of Interest

**Comorbidity**		**ICD-9-CM Code**
**CKD**	Chronic kidney disease (Depressed GFR)[1]	DX 016.00, 016.01, 016.02, 016.03, 016.04, 016.05, 016.06, 095.4, 189.0, 189.9, 223.0, 236.91, 249.40, 249.41, 250.40, 250.41, 250.42, 250.43, 271.4, 274.10, 283.11, 403.01, 403.11, 403.91, 404.02, 404.03, 404.12, 404.13, 404.92, 404.93, 440.1, 442.1, 572.4, 580.0, 580.4, 580.81, 580.89, 580.9, 581.0, 581.1, 581.2, 581.3, 581.81, 581.89, 581.9, 582.0, 582.1, 582.2, 582.4, 582.81, 582.89, 582.9, 583.0, 583.1, 583.2, 583.4, 583.6, 583.7, 583.81, 583.89, 583.9, 584.5, 584.6, 584.7, 584.8, 584.9, 585, 585.1, 585.2, 585.3, 585.4, 585.5, 585.6, 585.9, 586, 587, 588.0, 588.1, 588.81, 588.89, 588.9, 591, 753.12, 753.13, 753.14, 753.15, 753.16, 753.17, 753.19, 753.20, 753.21, 753.22, 753.23, 753.29, 794.4 (any DX on the claim)
**CVD**	**(congestive heart failure, stroke, acute myocardial infarction, peripheral vascular disease)**	
	Congestive heart failure[1]	DX 398.91, 402.01, 402.11, 402.91, 404.01, 404.11, 404.91, 404.03, 404.13,<br>404.93, 428.0, 428.1, 428.20, 428.21, 428.22, 428.23, 428.30, 428.31, 428.32,<br>428.33, 428.40, 428.41, 428.42, 428.43, 428.9 (any DX on the claim)
	Stroke / Transient Ischemic Attack[1]	DX 430, 431, 433.01, 433.11, 433.21, 433.31, 433.81, 433.91, 434.00, 434.01,<br>434.10, 434.11, 434.90, 434.91, 435.0, 435.1, 435.3, 435.8, 435.9, 436, 997.02 (any DX on the claim)
	Acute myocardial infarction[1]	DX 410.01, 410.11, 410.21, 410.31, 410.41, 410.51, 410.61, 410.71, 410.81,<br>410.91 (ONLY first or second DX on the claim)
	Peripheral artery disease (Suggestions from CCS)[2]	

	114 Peripheral and visceral atherosclerosis	440.0, 440.1, 440.2, 440.20, 440.21, 440.22, 440.23, 440.29, 440.4, 440.8, 440.9, 443.9, 557.0, 557.1, 557.9
	115 Aortic; peripheral; and visceral artery aneurysms	441.0, 441.00, 441.01, 441.02, 441.03, 441.1, 441.2, 441.3, 441.4, 441.5, 441.6, 441.7, 441.9, 442.0, 442.1, 442.2, 442.3, 442.81, 442.82, 442.83, 442.84, 442.89, 442.9, 443.21, 443.22, 443.23, 443.24, 443.29, 447.70, 447.71, 447.72, 447.73
	116 Aortic and peripheral arterial embolism or thrombosis	444.0, 444.01, 444.09, 444.1, 444.21, 444.22, 444.81, 444.89, 444.9, 445.01, 445.02, 445.81, 445.89
	117 Other circulatory disease	443.0, 443.1, 443.81, 443.82, 443.89, 446.0, 446.1, 446.2, 446.20, 446.21, 446.29, 446.3, 446.4, 446.5, 446.6, 446.7, 447.0, 447.1, 447.2, 447.3, 447.4, 447.5, 447.6, 447.8, 447.9, 448.0, 448.1, 448.9, 458.0, 458.1, 458.8, 458.9, 459.0, 459.89, 459.9, 785.9, 794.30, 794.31, 794.39, 796.2, V125, V1250, V1253, V1254, V1259, V151, V421, V432, V4321, V4322, V434, V717
**Asthma**	Asthma[1]	DX 493.00, 493.01, 493.02, 493.10, 493.11, 493.12, 493.20, 493.21, 493.22, 493.81, 493.82, 493.90, 493.91, 493.92, (any DX on the claim)
**Depression**	Depression[1]	DX 296.20, 296.21, 296.22, 296.23, 296.24, 296.25, 296.26, 296.30, 296.31, 296.32, 296.33, 296.34, 296.35, 296.36, 296.51, 296.52, 296.53, 296.54, 296.55, 296.56, 296.60, 296.61, 296.62, 296.63, 296.64, 296.65, 296.66, 296.89, 298.0, 300.4, 309.1, 311 (any DX on the claim)

CCS= Clinical Classifications Software; DX=diagnosis; HCUP= Healthcare Cost and Utilization Project; GFR=glomerular filtration rate.

[1]Chronic Conditions Data Warehouse. Condition Categories. http://www.ccwdata.org/web/guest/condition-categories. Accessed July 10, 2013.

[2]HCUP Clinical Classifications Software for ICD-9-CM. Healthcare Cost and Utilization Project. 2013. Agency for Healthcare Research and Quality, Rockville, MD. www.hcup-us.ahrq.gov/toolssoftware/ccs/ccs.jsp. Accessed June 17, 2013.
