# Supplementary material for: Benefits of applying a proxy eligibility period when using electronic health records for outcomes research: a simulation study
Source: BMC Res Notes. 2015 Jun 9;8:229. doi: 10.1186/s13104-015-1217-6 (PMC4467672; doi:10.1186/s13104-015-1217-6)
Supplement: Supplementary file 2 — Additional file 2: Figure S1. Root-mean-square error in parameter estimates. Box plots of root-mean-square error in selected parameter estimates by percentage of individuals with missing data for the restricted analysis and the unrestricted analysis. Panel a. Percentage of patients with cardiovascular disease, b. Percentage of patients experiencing shortness of breath, c. Mean number of shortness of breath occurrences per patient, d. Percentage of patients prescribed ICS/LABA, e. Mean number of ICS/LABA prescriptions per patient, f. Percentage of patients with an exacerbation, g. Mean number of exacerbations per patient, h. Percentage of patients with a hospitalization, and i. Mean number of hospitalizations per patient. [file 13104_2015_1217_MOESM2_ESM.pdf]

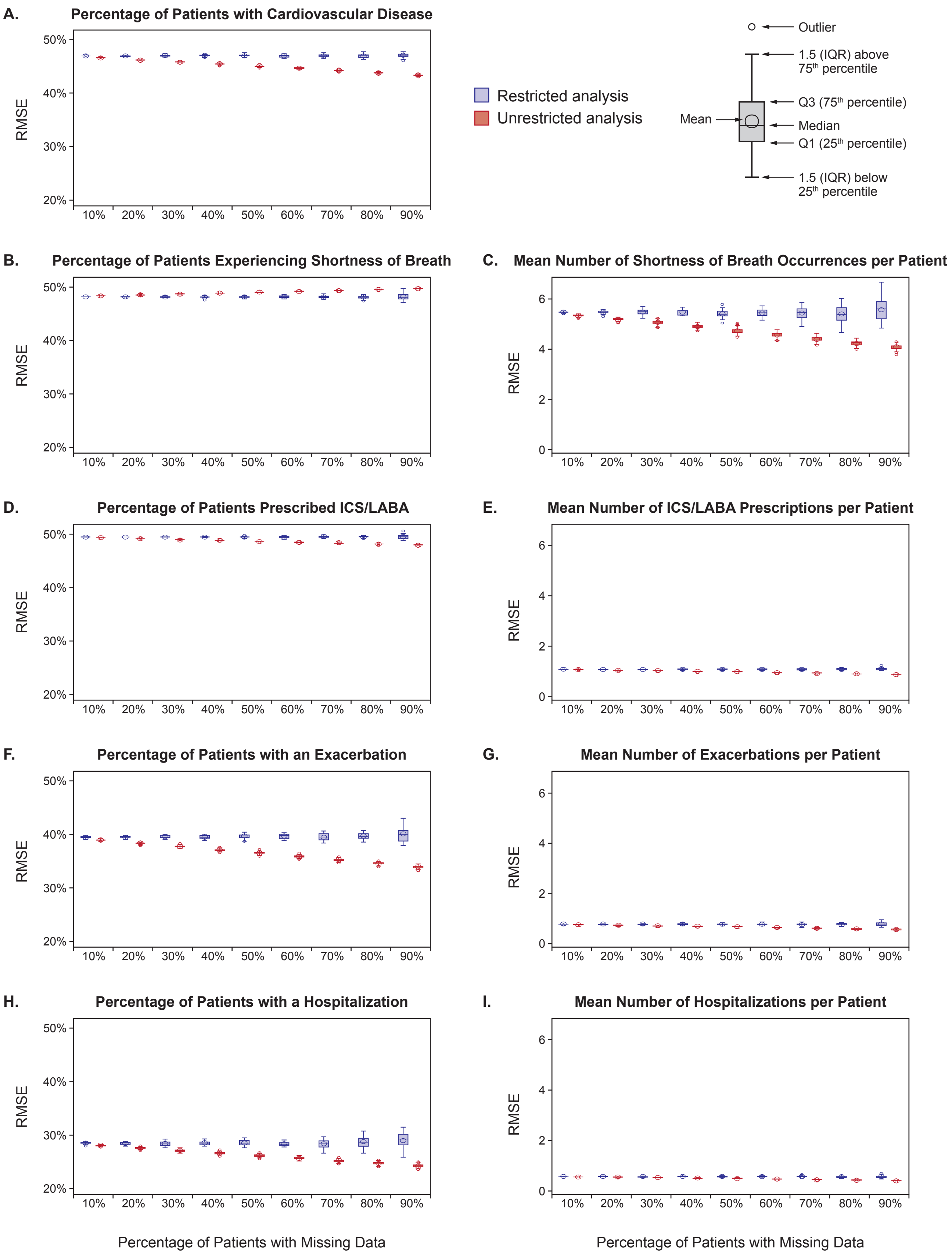
A.
Percentage of Patients with Cardiovascular Disease
RMSE
50%
40%
30%
20%
10%
20%
30%
40%
50%
60%
70%
80%
90%
Restricted analysis
Unrestricted analysis
Outlier
1.5 (IQR) above 75th percentile
Q3 (75th percentile)
Mean
Median
Q1 (25th percentile)
1.5 (IQR) below 25th percentile
B.
Percentage of Patients Experiencing Shortness of Breath
C.
Mean Number of Shortness of Breath Occurrences per Patient
D.
Percentage of Patients Prescribed ICS/LABA
E.
Mean Number of ICS/LABA Prescriptions per Patient
F.
Percentage of Patients with an Exacerbation
G.
Mean Number of Exacerbations per Patient
H.
Percentage of Patients with a Hospitalization
I.
Mean Number of Hospitalizations per Patient
0
2
4
6
Percentage of Patients with Missing Data
Percentage of Patients with Missing Data
